# Does Multi-Component Strategy Training Improve Calculation Fluency Among Poor Performing Elementary School Children?

**DOI:** 10.3389/fpsyg.2018.01187

**Published:** 2018-07-11

**Authors:** Tuire K. Koponen, Riikka Sorvo, Ann Dowker, Eija Räikkönen, Helena Viholainen, Mikko Aro, Tuija Aro

**Affiliations:** ^1^Faculty of Education and Psychology, University of Jyväskylä, Jyväskylä, Finland; ^2^Niilo Mäki Instituutti, Jyväskylä, Finland; ^3^Department of Education, University of Jyväskylä, Jyväskylä, Finland; ^4^Department of Experimental Psychology, University of Oxford, Oxford, United Kingdom; ^5^Department of Psychology, University of Jyväskylä, Jyväskylä, Finland

**Keywords:** intervention, calculation fluency, calculation strategies, derived fact, mathematical learning difficulties

## Abstract

The aim of the present study was to extend the previous intervention research in math by examining whether elementary school children with poor calculation fluency benefit from strategy training focusing on derived fact strategies and following an integrative framework, i.e., integrating factual, conceptual, and procedural arithmetic knowledge. It was also examined what kind of changes can be found in frequency of using different strategies. A quasi-experimental design was applied, and the study was carried out within the context of the school and its schedules and resources. Twenty schools in Finland volunteered to participate, and 1376 children were screened in for calculation fluency problems. Children from second to fourth grades were recruited for the math intervention study. Children with low performance (below the 20th percentile) were selected for individual assessment, and indications of using counting-based strategies were the inclusion criteria. Altogether, 69 children participated in calculation training for 12 weeks. Children participated in a group based strategy training twice a week for 45 min. In addition, they had two short weekly sessions for practicing basic addition skills. Along with pre- and post-intervention assessments, a 5-month follow-up assessment was conducted to exam the long-term effects of the intervention. The results showed that children with dysfluent calculation skills participating in the intervention improved significantly in their addition fluency during the intervention period, showing greater positive change than business-as-usual or reading intervention controls. They also maintained the reached fluency level during the 5-month follow-up but did not continue to develop in addition fluency after the end of the intensive training program. There was an increase in fact retrieval and derived fact/decomposition as the preferred strategies in math intervention children and a decrease of the use of counting-based strategies, which were the most common strategies for them before the intervention. No transfer effect was found for subtraction fluency.

## Introduction

Arithmetic calculation is a basic academic skill that, along with reading and writing skills, forms the foundation for academic learning and practical skills of daily life. While there are some national and cultural differences, some studies suggest that approximately 20% of people struggle with basic numerical skills (e.g., Bynner and Parsons, 1997). Studies in several countries suggest that about 5–7% of the population have severe specific mathematical learning difficulties (MD) (Shalev et al., 2005; Butterworth et al., 2011; Geary et al., 2012), although the figure depends on the exact criteria used for diagnosing MD (Kaufmann et al., 2013). In general, the term Mathematical Learning Difficulty (MD) is used broadly to describe a wide variety of deficits in math skills, such as problems in the estimation and processing of quantity and in using the mental number line, in transcoding between number words, digits and quantities or problems in understanding the Base-10 number system or fluently solve simple arithmetic problems and instead use immature counting strategies (e.g., Gersten et al., 2005). It has been proposed that arithmetical fact retrieval deficit resistant to instructional intervention might be a useful diagnostic indicator of arithmetical forms of MLD (Geary, 2004). In the present study we will focus on these arithmetic dysfluency problems.

Difficulties in arithmetic can have serious long-term consequences for later school achievement and limit one’s societal and occupational opportunities in adult life (Bynner and Parsons, 1997; Parsons and Bynner, 2005). Individuals with numeracy difficulties tend to leave school early, frequently without qualifications, and have more difficulty than those without such difficulties in getting and maintaining full-time employment (Bynner and Parsons, 1997; Parsons and Bynner, 2005). Gross et al. (2009) estimated that mathematics learning problems reduce an individual’s earnings by at least 10%, even after controlling for socio-economic status and other factors. Effective tools for support should be available at schools to provide adequate basic skills and to diminish later difficulties in basic mathematical skills, and thus, prevent long-lasting negative impacts.

Dysfluency in arithmetic calculation, i.e., difficulty in fact retrieval is the most typical feature of MDs. Children with dysfluency problems often rely on slow and error-prone counting strategies, such as *counting all* or *counting on from the first number* (Geary, 2004). They show problems in shifting from immature counting strategies to more advanced strategies, such as direct and fast *fact retrieval*, *decomposing* the problem into smaller facts (7 + 6 → 7 + 3 = 10, 10 + 3 = 13), or *deriving* unknown arithmetical facts from known facts (7 + 6 → 6 + 6 = 12 → 7 + 6 = 12 + 1 = 13), despite several years of formal schooling. The differences in math performance between typically performing children and children with MDs can be striking. Even young primary school children can often retrieve answers from memory or derive and predict unknown arithmetical facts from known facts without direct teaching (Dowker, 1998, 2014; Canobi, 2005), whereas children with difficulties may not learn to use these more advanced strategies despite practicing arithmetic at school for several years and despite having a normal cognitive capacity. Previous intervention research aimed at enhancing calculation fluency in children with MDs has generally focused either on training fact retrieval itself or more efficient counting-based strategies, such as counting on from the largest number, (e.g., Christensen and Gerber, 1990; Tournaki, 2003; Fuchs et al., 2006), and thus the effectiveness of training MD children in derived fact and decomposition strategies remains unclear.

The development of calculation fluency is a multidimensional process. According to the *overlapping waves theory* (Siegler, 1996), one dimension influencing the development of calculation fluency is the frequency of using different strategies: during the typical development of calculation, more efficient strategies (such as fact retrieval and deriving/decomposing) become more dominant. According to this view, difficulties in calculation fluency involve the infrequent use of efficient calculation strategies and the frequent use of slow and error-prone counting-based strategies. Difficulties in calculation fluency and in making the shift to more frequent use of more efficient strategies can stem from several sources. First, it has been suggested that rapid *access to long-term memory* is central for the ability to retrieve arithmetical facts from memory, and that difficulties in this area constitute the key deficit underlying calculation dysfluency among children with MDs, making it difficult for them to use the most efficient strategies. This deficit is particularly marked regarding learning multiplication tables, which is the arithmetical operation mostly relying on arithmetical fact retrieval, and it is also required for fluent addition and subtraction.

The second key deficit might be related to *conceptual knowledge*, which enables individuals to determine the answer to an unknown problem using some known fact, i.e., using derived fact strategies and/or dividing the problem into smaller sums that are easier to solve or retrieve (decomposition), and thus can provide effective back-up strategies when fact retrieval is not possible. Dowker (2009) has suggested that use of these derived fact and decomposition strategies might be an indication of the extent to which children have an explicit understanding of the connections between individual number facts and/or between different arithmetical operations. Thus, a lack of conceptual understanding might be one reason children with MDs do not typically use the more advanced strategies but rely mostly on slower counting-based strategies, such as to start counting from the first addend in the problem *(COF/Counting on from the first number)* rather than the more sophisticated strategy, i.e., *Counting min strategy*, where counting starts from the larger addend. The third deficit is related to the mastery of rules and calculation procedures, i.e., *Procedural knowledge* (Geary, 1993), which means knowing how to use certain arithmetic strategies, such as “borrowing: in subtraction.”

This classification of deficits is in line with the theory that arithmetical knowledge consists of at least three different types of knowledge: factual, conceptual, and procedural (Girelli et al., 2002). Deficit in one type of knowledge might be compensated when using other knowledge as well as by learning how to integrate these knowledge. Difficulty in retrieving arithmetical facts from memory is one of the most consistent findings in the MD literature (e.g., Geary, 1993; Jordan et al., 2003; Cirino et al., 2007; Geary et al., 2007), and this difficulty is known to be rather persistent. Thus, including two other components, procedural and conceptual knowledge, for training, in addition to fact retrieval, could contribute to the development of compensatory mechanisms for children with difficulties in arithmetical calculation (Girelli et al., 2002).

In recent times, a wide variety of educational interventions have been developed for helping children with difficulties of varying severity in mathematics (Chodura et al., 2015; Dowker, 2017). They have targeted a wide variety of components and subcomponents of arithmetic and have been flexibly adapted to individual children, e.g., Catch Up Numeracy TM (Dowker and Sigley, 2010; Holmes and Dowker, 2013) and Numbers Count (Torgerson et al., 2011). However, it is still true to say that most educational interventions thus far have targeted just one component, most commonly factual knowledge trained by drilling (e.g., Christensen and Gerber, 1990; Hasselbring et al., 1988; Fuchs et al., 2006). Some more recent studies have, however, compared two or more interventions focusing on different components, and thus using different methods of training, e.g., drilling (factual knowledge) with procedural strategy training (with or without conceptual knowledge) versus more general procedural training with multi-digit numbers (Powell et al., 2009), or drilling alone versus a procedural strategy training alone versus the combination of approaches (Woodward, 2006; Fuchs et al., 2008; for review, see Fuchs et al., 2010).

Findings as to the effectiveness of these approaches are mixed. Some of the studies suggest that children with MDs benefit more from strategy instruction or a combination of strategy training and drilling instead of instruction through pure drill and practice, which targets only factual knowledge (Tournaki, 2003). In the study by Tournaki (2003), single-digit addition facts were taught through strategy instruction as well as drill and practice. The results showed that second graders with MDs benefited more from strategy instruction than from instruction through drill and practice (Tournaki, 2003), whereas typically developing controls improved significantly both in the strategy and the drill-and-practice conditions compared to the control condition. However, these two intervention conditions also differed regarding feedback, in that immediate feedback was provided in the strategy condition and delayed feedback in drill-and-practice conditions, so that is difficult to separate the effects of the differences in feedback from those in training (see Powell et al., 2009). In an intervention study by Woodward (2006) with a group of fourth graders (9–10 year olds) with a wide ability range in arithmetic, a combination of strategy training and drilling on facts led to greater improvement in calculation fluency than drilling alone. In contrast, Powell et al. (2009) did not find any differences in post-test performance between children with MDs who received just fact retrieval training and those who received a combination of fact retrieval and strategy instruction among children with MDs. Both intervention groups performed significantly better at the post-test than a business-as-usual control group.

The concept of integrating all three kinds of arithmetical knowledge has been applied in few single-case intervention studies. Case studies with adult (Girelli et al., 2002) and with child (Koponen et al., 2009) have suggested that if arithmetical fact retrieval is severely impaired and resistant to intervention, a better way of improving children’s calculation skills might be to train them in more efficient calculation strategies that rely on procedural and conceptual knowledge. The main principle of both studies was that rather than training children in arithmetical facts by rote learning, the aim was to enable them to use conceptual and procedural knowledge to construct calculation strategies based on meaningful relationships between the known and unknown arithmetical facts. This is important, both because derived fact strategies are themselves an important aspect of arithmetical reasoning (Dowker, 1998, 2014; Canobi, 2005; Star and Rittle-Johnson, 2008) and because children with difficulties in fact retrieval may be able to use such strategies to compensate. Although rigid counting-based strategy use characterizes many children with MDs, some studies suggest that the ability to use derived fact strategies is a relative strength for some low attainers in arithmetic (Russell and Ginsburg, 1984; Dowker, 2009); it may be possible to capitalize on this in enabling them to develop and use compensatory strategies. In Koponen et al. (2009), single-case study, a child was trained to use known arithmetical facts to derive other facts by comparing the magnitude of numbers presented in one arithmetical problem to those of the other problem. He was enabled to determine, based on this comparison, and his previous knowledge of arithmetical operations and principles, how the answers of the two arithmetical problems differed in magnitude (e.g., 5 + 5 = 10, 5 + 6 = ?). The procedural training that he received was linked to his existing conceptual knowledge of numbers and arithmetical operations as well as some familiar arithmetical facts, such as 5 + 5 = 10.

Because there are only a few studies, mostly focusing on single cases, more evidence is needed regarding the effect of strategy intervention integrating the three types of arithmetical knowledge. Besides individually tailored remediation, there is a need for intervention tools and programs that can be effectively applied in small groups or even in classrooms to support calculation fluency among children to whom curriculum-based instruction and training at school is not sufficient to provide adequate calculation skills. This would contribute to such an intervention program becoming sustainable in a school long term, independently of a concurrent research program.

Another gap in the existing intervention literature is that many previous studies focusing specifically on strategies have focused on a rather limited set of strategies emphasizing those usually learned at early phase of typical strategy development. For example, in a study by Tournaki (2003), strategy training included teaching the *minimum addend* strategy, in which the student determines the larger addend and counts on from that cardinal value the number units specified by the smaller addend (e.g., 2 + 6, students start from 6 and adds two more). Fuchs et al. (2009) carried out an intervention in which children practiced n + 0, n + 1, n + 2 strategies utilizing counting sequence and number knowledge, and although the doubles (2 + 2; 6 + 6 etc.) were trained as well, the focus was on *“know it or count it.”* There have been rather few intervention programs emphasizing alternative calculation strategies, such as derived fact strategies, among children with poor calculation fluency. There are, however, several studies of interventions involving training in derived fact strategy use, which have tended to yield positive short-term results, but most such studies have either been embedded in practice rather than research and have, for example, lacked control groups (Thornton, 1978; Steinberg, 1985; Adetula, 1996; Askew et al., 2001) or have included derived fact strategy training as just one of many components of an intervention program (Dowker and Sigley, 2010; Holmes and Dowker, 2013; Bakker et al., 2015), making it hard to assess the specific impact of derived fact strategy training.

One study that did compare derived fact strategy training with procedural training was carried out by Caviola et al. (2016). They divided 219 third and fifth graders into three approximately equal groups: a computer-based (derived fact) strategic training group in mental addition, a procedural training group in mental addition, and a business-as-usual control group. Both forms of training had positive effects on addition post-tests, with the strategic training being more effective with the third graders, and the procedural training with the fifth graders. This study did not, however, focus on children with MDs.

Moreover, in previous studies calculation outcome measures have mainly involved calculation fluency and accuracy, not the frequency of use in different strategies. Thus, it does not allow for concluding which type of intervention promotes the use of which strategies (Fuchs et al., 2010). Finally, most of the above-mentioned intervention studies have not examined whether the intervention effect is maintained over time, i.e., whether training enhances the learning only temporarily or whether there are long-term benefits.

### Present Study

The present study extends the previous intervention research in math by examining whether children with poor calculation fluency benefit from derived fact strategy training based on an integrative framework (i.e., integrating factual, conceptual, and procedural knowledge training) administered at a school setting in small groups. The long-term benefits of the intervention were assessed 5 months after the intervention ended. The development of the Math intervention group was compared with two different kinds of control groups, one receiving similar kinds of intensive support provided by a special education teacher and implemented in small groups but in a different context (reading intervention group). Another control group consisted of classmates, who were performing the “next poorest” in the classroom, matched for gender (if possible) and who had the same classroom teacher as the Math intervention group and received business-as-usual instruction at school. Both calculation fluency and changes in the frequency of using different kinds of strategies were assessed. The specific research questions were:

(1) Does explicit strategy training integrating factual, conceptual, and procedural knowledge improve the calculation fluency of addition among children with poor calculation fluency?
(2) Does the calculation fluency development of the explicit strategy training group differ from that of a control group receiving a similarly intensive reading intervention (controlling for additional instructional attention and peer group support) or from the development of business-as-usual classmate controls with low performance in calculation fluency?
(3) Does the explicit strategy training integrating factual, conceptual, and procedural knowledge also change the frequency of use in different strategies?

## Materials and Methods

### Participants

This study was part of a longitudinal *Self-efficacy and Learning Disability Intervention* research project (SELDI; 2013–2015) focusing on elementary school children’s self-beliefs, motivation, and reading and math fluency skills, and in support of children with reading or math difficulties. The data for the present study were collected between November 2013 and October 2014. A total of 20 schools in urban and semi-urban areas in Central and Eastern Finland volunteered to participate, from which the classes and children were recruited for this study. Written consent was obtained from the guardians of the participants. The research procedure was evaluated by the University of Jyväskylä Ethical Committee.

The original sample consisted of 1,327 children (638 girls, 689 boys) from grades 2 to 5. Of the participants, 178 (13.41% of the original sample) were second graders (*M*_age_ = 8.35 years, *SD* = 0.32 years), 471 (35.49%) were third graders (*M*_age_ = 9.34 years; *SD* = 0.31 years), 383 (28.86%) were fourth-graders (*M*_age_ = 10.40 years; *SD* = 0.35 years), and 295 (22.23%) were fifth graders (*M*_age_ = 11.39 years; *SD* = 0.36 years). A calculation strategy training was provided for children from second to fourth grades.

A quasi-experimental design was applied, as the school, classes, and teachers volunteered to participate, written consent from parents was required to participate, and the study was carried out within the context of the school and its schedules and resources. Screening was conducted according to both reading and calculation fluency, and volunteer teachers were randomized to have either reading or arithmetic training group with or without specific self-efficacy feedback. Approximately half of the children participating in the Math intervention received self-efficacy feedback, following the intervention manual, and the other half received the usual feedback given by special education teacher also providing the strategy training. Both groups had identical strategy training. These two intervention groups were balanced according to the calculation fluency in the pretest. The two groups neither differed in addition fluency at any assessment point nor in development (*p* < 0.05) and were thus treated as a unitary group in the present study.

#### Screening Procedure for Intervention

Screening for the calculation strategy intervention included two steps. First, all participants from the original sample were assessed in terms of their calculation fluency using group-administered timed calculation tasks (Koponen and Mononen, 2010a, unpublished). Children from grades 2 to 4 whose performance was at or below the 20th percentile in the calculation fluency task were then selected for individual assessment. Individual assessment included 20 single-digit addition items (2 + 8, 5 + 4, 9 + 6, 7 + 3 etc.) presented one by one in a game-like situation. Children were asked to respond as quickly as possible to each item. Only for correct responses given within 3 s, a point was scored. Inclusion criteria for the intervention were that children showed dysfluency, both in the group-administered calculation fluency task (i.e., performance at or below the 20th percentile) and in the individual assessment situation requiring fast fact retrieval or the efficient use of back-up strategies (slow or incorrect response at least 30% of the simple addition items). Altogether, 69 children met this selection criteria and were included in the present analyses. An additional six children with low calculation fluency, but who did not meet the selection criterion, participated in the Math intervention for practical reasons (i.e., to be able to form a group) but were not included in the analyses.

#### Control Groups

In the present study, the development of the Math intervention group during the baseline, intervention, and follow-up periods was contrasted with the development of the reading intervention controls and the classmate controls. To form the classmate control groups (*N* = 69), one child from the class of each participant of the Math intervention was selected based on having the next-lowest addition fluency score.

Classmate controls were matched for gender (when possible), and they received business-as-usual support, including special education usually provided in the school. The reading intervention group consisted of children with reading fluency deficits who received the intervention as part of the SELDI project in small groups during the same period (*N* = 85; for details, see Aro et al., in press).

#### Intervention Design and Procedure

We applied an intervention design with two pre-, one post-, and one follow-up assessment. Pre-intervention assessments were conducted in November and January. The 12-week-long interventions started in the end of January. A post-intervention assessment was conducted right after the intervention ended in April, and a follow-up assessment 5 months after ending the intervention in the end of September or in the beginning of October. As an exception, the forced fact retrieval and arithmetic fluency tasks were not repeated in January at the second pre-intervention assessment, and strategy use in free-choice condition was assessed at second but not the first pre-intervention assessment.

All calculation fluency tasks together with reading fluency tasks, non-verbal reasoning tasks, self-efficacy and other questionnaires were administered in groups and conducted during three assessment sessions (30–45 min each) at pre1-, post- and follow-up assessments. At the second pre-intervention assessment shortened assessment battery, including addition and subtraction fluency tasks, was administered during one group assessment session. Group assessment was administered before individual assessment at each time point.

### Measures

#### Calculation Fluency Measures

Basic addition and arithmetic fluency were assessed using one individually administered game-like assessment task administered individually, as well as three group paper-and-pencil tests with time limits.

The individual game-like assessment used a *no-choice technique to assess* addition fluency. The children were shown a card with an addition problem on it and were required to answer correctly less than in 3 s to win the card. For the sake of simplicity, we call this test the *forced fact retrieval task* and the outcome variable *fact retrieval ability*, as has been done in several previous studies (Russell and Ginsburg, 1984; Siegler and Shrager, 1984; Jordan and Montani, 1997; Geary et al., 2000, 2012; Jordan et al., 2003). However, at the same time we must accept the fact that other fast back-up strategies are also possible despite the short time allowed for solving the problem, e.g., derived fact strategies. As a *screening and near transfer task* children were given a 2-min group test of addition fluency (Koponen and Mononen, 2010a, unpublished), which consisted of 120 items with addends smaller than 10. As a *far transfer task, children* were given a similar subtraction test (Koponen and Mononen, 2010b, unpublished) consisting of 120 items with answers in the range of 1 to 9 and 2-min time limit. Another *far transfer task* was the three-minute Basic Arithmetic test (Aunola and Räsänen, 2007), which consists of 30 single-digit and multidigit addition, subtraction, division, and multiplication items. In each test, one point was given from all correctly solved items, and the sum score was counted for each test. Correlation between addition, subtraction and arithmetic tasks in original sample varied from 0.74 to 0.85.

*Strategy use in a free-choice condition* was assessed with 12 addition items in a similar manner as in the forced fluency task with the exception that children were instructed to solve each addition item in a way that is best for them, i.e., the way that will get the correct answer as quickly as possible. The response time was measured, strategy use was observed, and children were asked to describe/show how they calculated if this was unclear. Strategies were classified into four groups. If a child answered correctly within 3 s and without any signs of using counting, the strategy was classified as *fact retrieval*. If a child’s response time was over 3 s but no signs of using a counting strategy were observed or reported or the child reported that he/she used 10 pairs, doubles, or some other known arithmetical fact as a help or used a decomposition strategy, the strategy was classified as *derived fact/decomposition*. If the child’s response time was 3 s or more and if the child reported or demonstrated the use of counting, the strategy was classified as mental *counting* or *counting aloud*, depending on whether s/he produced number words silently or aloud.

### Background Measures

Non-verbal reasoning was assessed in a group situation using Raven’s Colored Progressive Matrices (CPM; Raven et al., 1999). The CPM comprises 36 items divided into three sets of 12 (set A, Ab, and B). Within each set, items are ordered in terms of increasing difficulty. Additionally, vocabulary was assessed individually using the Vocabulary subtest from the Wechsler Intelligence Scale for Children-IV (WISC-IV; Wechsler, 2010) with Finnish normative data. In this task words of increasing difficulty are presented orally, and children are required to define the words. According to test manual Cronbach’s alpha for 8–11 years old varied from 0.83 to 0.87. Visuo-spatial skills were assessed using the Block Design subtest of the WISC-IV (Wechsler, 2010). In this test, the individual is presented with identical blocks with surfaces of red, surfaces of white, and surfaces that are half red and half white. Using an increasing number of these blocks, the individual is required to replicate a pattern that the examiner presents to them—first as a physical model, and then as a two-dimensional picture. The number of blocks required to match the presented models increases, and the patterns become increasingly difficult to visually dissect into components. According to test manual Cronbach’s alpha for 8 to 11 years old varied from 0.73 to 0.76.The standardized scores of each test are presented in **Table 1**.

**Table 1:** Descriptive statistics of background variables for the math intervention, reading intervention and business as usual controls.

	Math intervention	Reading intervention	Controls
	
*N*	69	85	69
Gender (boys%)	48%	66%^∗^	49%
Age (*M*)	113.51	123.99^∗∗∗^	113.21
*SD*	*10.65*	*11.48*	*12.5*
Raven^a^ (*M*)	8.74	9.04	9.67
*SD*	*3.81*	*3.27*	*3.00*
Block design^a^	8.65	9.16	NA
*SD*	*3.22*	*3.15*	NA
Vocabulary^a^	7.89	7.65	NA
	2.75	3.34	NA

*^a^Standard score (Mean = 10, SD = 3). NA, not available. ^∗^p < 0.05, ^∗∗∗^p < 0.001.*

### Intervention Program

In the present intervention study, a shortened version of the SELKIS intervention program (Koponen et al., 2011) was used. This program focuses on derived fact strategy training and aims at helping children to discover more efficient calculation strategies using their existing knowledge of number sequences, number concepts, and arithmetical facts (conceptual knowledge). Children participated in the Strategy training group sessions twice a week for 45 min. The number of participants in the groups varied between 4 and 6. In addition, they had two short weekly Gaming sessions for practicing basic addition skills by playing math games and got a worksheet for homework including similar kinds of additions practiced during strategy sessions.

#### Strategy Training Group Sessions

Addition strategies were trained twice a week in group sessions conducted by special education teacher following the intervention manual. The contents and order of strategy training is presented in **Table 2**. Each session started with checking the homework and followed by instruction sessions, exercises, and closing. Each session consisted of one or two, about 10–15 min’ long, strategic instruction sessions as well as of short games and exercises. During the instruction teacher modeled and discussed with children about the magnitude relations between numbers and how counting sequence and addition are linked with this knowledge of number relations (two steps forward in counting sequence – number that is two larger – *x* + 2). Moreover, children were instructed to pay attention and compare how arithmetical facts are related according magnitude (5 + 5 and 6 + 5, six is one more than five, six and five makes one more than five and five). These discussions aimed at guiding the children to discover new strategies based on conceptual understanding. Intervention program manual instructed teachers to encourage children to verbalize their thinking and strategies as well as to point out that use of several strategies is possible and each child should find the fastest strategies for him or herself. After instruction sessions children practiced calculation strategies by playing familiar games embedded with arithmetical contents, such as a board game with doubles and doubles +1, Bingo, card games with ten pairs.

**Table 2:** Contents of math intervention.

Session 1:	Starting session
Sessions 2–4:	Rules for adding one or two (*N* + 1 and *N* + 2) as well as commutativity principle for addition (*a* + *b* = *b* + *a*)
Sessions 5–6:	Add to five (5+, 1/2/3/4/5). Decomposing numbers 6–9 to 5 and *x*. Verbalizing “*five and two makes seven.”*
Sessions 7–10:	10-pairs and 10-pairs plus 1 (deriving answer by using 10-pairs, 5 + 6 → 5 + 5 = 10 and 5 + 6 “is one more” 11);
Session 11:	Add to 10 (structure of numbers 11–19). Verbalizing “10 and 2 makes 12.”
Session 12:	Rehearsal
Sessions 13–14:	Use the structure of five when solving sums with numbers from 5 to 9 (6 + 7 = 5 + 1 + 5 + 2 = 10 + 3)
Sessions 15–18:	Doubles and doubles plus 1 (deriving answer by using doubles, 7 + 6 → 6 + 6 = 12 and 7 + 6 “is one more” 13)
Sessions 19–22:	Add to 9 or 8 (deriving answer by using sums including number 10; 10 + 7 = 17, 9 + 7 is one less and 8 + 7 is two less)
Session 23:	Rehearsal
Session 24:	Ending session

#### Gaming Sessions

Short game-like practicing sessions were arranged twice a week each lasting about 15 min. The Gaming sessions were organized and instructed by school assistant or classroom teacher who followed the intervention manual. During these sessions children played games that were already introduced during the Strategy training sessions (card games, board games, etc.) and the aim was to provide repetitions in using addition strategies and achieve fluency. After each session children got a marking (sticker or stamp) to their “game chart.”

### Teacher Training and Fidelity

Before the intervention periods, researchers instructed all participating teachers on how to implement the intervention program and provided them with detailed session-by-session manuals. Two 3-h-long training sessions were organized including the theory of calculation fluency development as well as how to implement intervention in practice using the program manual. After the third intervention session, researchers called to each teacher to ensure that manuals were followed, and main principals of the programs understood. Moreover, two meetings were arranged during the intervention to share experiences and ensure that all the teachers had common understanding of the key points. Teachers also filled a checklist type of diary, marking the completed intervention sessions and noting any exceptions in intervention activities or attendance of participants. There was altogether 128 activities within 24 strategy training sessions (introduction of strategies, games/exercises, starting and closing activities) and the average amount of activities completed by teachers without exceptions (e.g., didn’t have time enough) was 97%. The attendance percentage of individual children varied typically from 92 to 100% in a group meaning that in most of the groups one child was not absent more than 2 times out of 24 intervention sessions. However, there were four children that missed 4 out of 24 intervention sessions, one missed 5 and one 7. All these children were included in the analyses.

### Data Analyses

The mean values, mean standard scores and standard deviations of the background variables (Age, Raven’s CPM, Block Design, Vocabulary) were calculated. The differences between the Math intervention group and two control groups (Reading intervention controls and Business-as-usual controls) were analyzed by means of independent-samples *t*-tests. Gender differences were analyzed using Chi-square tests. The means and standard deviations for calculation fluency measured variables (fact retrieval, addition fluency, subtraction fluency, and arithmetic fluency) were calculated at each assessment point, and the mean differences between the math intervention group and classmate controls were tested using independent-samples *t*-tests. Differences between the Math intervention group and Reading group were analyzed using univariate analysis of covariance (ANCOVA) using age and gender as the covariate.

The intervention effect in the four outcome measures (fact retrieval, addition fluency, subtraction fluency, and arithmetic fluency) was first analyzed in the Math intervention group using univariate ANOVA for repeated measures (repeated-measures ANOVA) with *time* (pre-test1 vs. pre-test2 vs. post-test vs. follow-up) as a within-subject factor. The partial eta-square was calculated as a measure of effect size. In a second analysis, the progress of the Math intervention group was compared with that of the Business-as-usual controls, and *group* was added as a between-subjects factor. Because there were statistically significant differences in age and gender between the Reading and Math intervention groups, age and gender were used as covariates and univariate analysis of covariance for repeated measures (repeated-measures ANCOVA) as the analysis method. Where an interaction effect was found, planned contrasts on pre-, post-, and follow-up tests scores were conducted.

## Results

### Descriptive Statistics

The means and standard deviations of the age and standardized scores for the CPM, Block Design, and Vocabulary variables as well as percentage of boys in each group are presented in **Table 1**. There were significantly more boys than expected in the Reading intervention group and more girls in the Math intervention group [χ^2^(1) = 4.83, *p* < 0.05], and the children in the Reading group were on average older [*t*(141.81) = −5.60, *p* < 0.001]. As expected, there were no gender or age differences between the Math intervention group and the Business-as-usual controls, as the groups were originally matched for gender and grade [χ2(1) = 0.03, *p* > 0.05; *T*(134) = 0.26, *p* > 0.05]. Analyses showed that the Math intervention group did not differ significantly on the Raven’s Matrices test from either Business-as-usual controls [*t*(121.87) = −1.83, *p* > 0.05] or Reading intervention controls [*T*(122.23) = −1.78, *p* > 0.05]. There were no statistically significant differences between the Math and Reading intervention groups on either the Block Design test [*t*(146) = −0.96, *p* > 0.05] or the Vocabulary test [*tT*(144.92) = 0.63, *p* > 0.05] (data were not available for the Business-as-usual controls).

### Efficacy of the Intervention Among Children With Dysfluent Calculation Skills

The means and standard deviations of all calculation fluency measures (fact retrieval, addition fluency, subtraction fluency, and arithmetic fluency) for each group at each assessment point (pretest1, pretest2, post-test, follow-up) are presented in **Table 3**. The results of the repeated-measures ANOVAs for the math group are presented in **Table 4**. Statistically significant effects were found for time in all the calculation fluency tests. Calculation fluency showed favorable development among the Math intervention group throughout the entire study period in all four measured sub-skills. The effect sizes ranged from 0.24 to 0.65. The lowest level of improvement was found for subtraction and the highest for the forced fact retrieval and for addition fluency tasks.

**Table 3:** Performance at pretest, post-test and follow-up scores and mean differences.

Group	Math (*N* = 69)		*R* (*N* = 85)		*C* (*N* = 69)		Paired comparison	
	*M*	*SD*	*M*	*SD*	*M*	*SD*	Math vs. C	Math vs. R
**Fact retrieval**								
Pre	8.59	4.31	15.73	3.40	NA	NA		Math < R; *F*(1,139) = 73.66^∗∗∗a^
Post	15.59	2.80	17.05	2.81	NA	NA		Math < R; *F*(1,138) = 3.14 ns^a^
Follow-up	14.82	3.36	17.03	3.41	NA	NA		Math < R; *F*(1,132) = 7.10^∗∗a^
**Addition**								
Pre1	15.84	5.56	29.96	11.28	20.16	5.00	Math < C; *t*(136) = −4.82^∗∗∗^	Math < R; *F*(1,141) = 39.25^∗∗∗a^
Pre2	19.30	5.67	31.04	11.94	23.07	5.61	Math < C; *t*(126) = −3.78^∗∗∗^	Math < R; *F*(1,137) = 21.37^∗∗∗a^
Post	26.40	8.86	36.01	14.70	27.32	7.26	Math = C; *t*(126) = −0.64 ns	Math < R; *F*(1,140) = 4.13^∗a^
Follow-up	26.13	8.68	39.40	14.60	30.53	9.02	Math < C; *t*(126) = −2.81^∗∗^	Math < R; *F*(1,135) = 16.22^∗∗∗a^
**Subtraction**								
Pre1	14.01	5.54	25.52	9.99	18.39	6.34	Math < C; *t*(136) = −4.32^∗∗∗^	Math < R; *F*(1,142) = 33.86^∗∗∗a^
Pre2	14.76	6.12	23.27	10.10	17.41	6.62	Math < C; *t*(127) = −2.36^∗^	Math < R; *F*(1,137) = 15.39^∗∗∗a^
Post	16.15	5.64	26.99	10.78	21.97	6.85	Math < C; *t*(126) = −5.28^∗∗∗^	Math < R; *F*(1,140) = 25.56^∗∗∗a^
Follow-up	18.88	6.88	30.99	12.70	24.07	8.40	Math < C; *t*(126) = −3.84^∗∗∗^	Math < R; *F*(1,135) = 22.45^∗∗∗a^
**Arithmetic**								
Pre	8.13	3.59	13.33	4.08	10.71	4.71	Math < C; *t*(125.12) = −4.59^∗∗∗^	Math < R; *F*(1,139) = 30.29^∗∗∗a^
Post	10.50	4.30	13.85	5.12	12.68	3.72	Math < C; *t*(126) = −3.05^∗∗^	Math < R; *F*(1,140) = 5.27^∗a^
Follow-up	11.82	4.03	15.24	4.29	13.98	4.12	Math < C; *t*(126) = −3.00^∗∗^	Math < R; *F*(1,139) = 30.29^∗∗∗a^

*Math, math intervention group; R, reading intervention group; C, business-as-usual controls. ^a^age and gender as covariate. ^∗^p < 0.05, ^∗∗^p < 0.01, ^∗∗∗^p < 0.001.*

**Table 4:** Within-Group effect among math intervention group in calculation fluency across the time periods and task.

Task		WS effects	WS contrast		
			Baseline	Intervention	Follow-up
Forced fact retrieval	*F (df)*	118.8 (2, 126)	NA	194.6 (1, 63)	3.5 (1, 63)
	*sig.*	0.00	NA	0.00	0.07
	${\text{η}}_{\text{p}}^{2}$	0.65	NA	0.76	0.05
Addition fluency (2 min)	*F (df)*	64.1 (2, 119.5)^a^	33.0 (1.61)	58.0 (1, 61)	0.4 (1, 61)
	*sig.*	0.00	0.00	0.00	0.56
	${\text{η}}_{\text{p}}^{2}$	0.51	0.35	0.49	0.01
Subtraction fluency (2 min)	*F (df)*	20.0 (2.6, 162.7)^b^	4.23 (1.62)	4.1 (1, 62)	14.7 (1, 62)
	*sig.*	0.00	0.04	0.05	0.00
	${\text{η}}_{\text{p}}^{2}$	0.24	0.06	0.06	0.19
Arithmetic fluency (3 min)	*F (df)*	34.2 (2, 128)	NA	35.3 (1, 64)	5.9 (1, 64)
	*sig.*	0.00	NA	0.00	0.02
	${\text{η}}_{\text{p}}^{2}$	0.35	NA	0.36	0.08

*^a^Greenhouse-Geisser; ^b^Huynh-Feldt.*

Planned contrast was used to analyze the development of calculation fluency in the Math intervention group in more depth. The analysis of the calculation fluency tasks including addition (fact retrieval, addition fluency, and arithmetic fluency) indicated statistically significant development during the intervention period (*p* < 0.001, ${\text{η}}_{\text{p}}^{2}$ = 0.76; *p* < 0.001, ${\text{η}}_{\text{p}}^{2}$ = 0.49, *p* < 0.001, ${\text{η}}_{\text{p}}^{2}$ = 0.36, respectively). In addition, fluency task data were also available from the baseline period (pretest1–pretest2), showing significant improvement (*p* < 0.001, ${\text{η}}_{\text{p}}^{2}$ = 0.35). From the post-test to follow-up, significant improvement was found in arithmetic fluency (*p* < 0.05, ${\text{η}}_{\text{p}}^{2}$ = 0.08), but not in fact retrieval (*p* > 0.05, ${\text{η}}_{\text{p}}^{2}$ = 0.05) or addition fluency (*p* > 0.05, ${\text{η}}_{\text{p}}^{2}$ = 0.01). In subtraction fluency, the greatest improvement was during the follow-up (*p* < 0.001, ${\text{η}}_{\text{p}}^{2}$ = 0.19) after a very small but significant improvement during the intervention (*p* < 0.05, ${\text{η}}_{\text{p}}^{2}$ = 0.06).

### Group Differences in Calculation Fluency Development

First, we analyzed the *fact retrieval*, in which the data were available only for the Math and Reading intervention groups. Repeated-measures ANCOVA Statistically significant main effects of time were found, indicating that performance improved in both groups throughout the entire study period (**Tables 3**, **5**). More importantly, there was an interaction between time and group, which was further explored with planned contrasts.

**Table 5:** Within-group and between-group effects among math intervention and control groups in calculation fluency across the time periods and tasks.

Math and reading groups		WS effects		WS contrast Time^∗^Group		
		Time	Time^∗^Group	Baseline	Intervention	Follow-up
Forced fact retrieval	*F (df)*	3.97(1.82, 235.1)^b,c^	31.84 (1.7, 235.1)^b^	NA	55.84 (1, 127)	1.54 (1, 127)
	*sig.*	0.02	*p* < 0.001	NA	*p* < 0.001	0.22
	${\text{η}}_{\text{p}}^{2}$	0.03	0.20	NA	0.31	0.01
Addition fluency (2 min)	*F (df)*	1.83 (2.66, 343.52)^b,c^	4.03 (2.66, 343.52)^b^	0.93 (1, 129)	7.04 (1, 129)	8.71 (1, 129)
	*sig.*	0.14	0.01	0.34	0.01	0.01
	${\text{η}}_{\text{p}}^{2}$	0.01	0.03	0.01	0.05	0.06
Arithmetic fluency (3 min)	*F (df)*	1.05 (2, 264)^c^	5.16 (2, 264)	NA	8.96 (1, 132)	0.14 (1, 143)
	*sig.*	0.35	0.01	NA	0.01	0.71
	${\text{η}}_{\text{p}}^{2}$	0.01	0.04	NA	0.06	0.01

**Math and classmates groups**		**WS effects**		**WS contrast Time^∗^Group**		
		**Time**	**Time^∗^Group**	**Baseline**	**Intervention**	**Follow-up**

Addition fluency (2 min)	*F (df)*	93.07 (2.1, 236.1)^a^	4.28 (2.1, 236.1)^a^	2.92 (1, 112)	1.0 (1, 112)	1.0 (1, 112)
	*sig.*	0.00	0.01	0.09	0.04	0.00
	${\text{η}}_{\text{p}}^{2}$	0.45	0.04	0.03	0.04	0.11
Arithmetic fluency (3 min)	*F (df)*	57.64 (2, 234)	0.29 (2, 234)			
	*sig.*	.00	0.75			
	${\text{η}}_{\text{p}}^{2}$	0.33	0.00			

*^a^Greenhouse-Geisser; ^b^Huynh-Feldt; ^c^Age and gender as covariate.*

**FIGURE 1:**
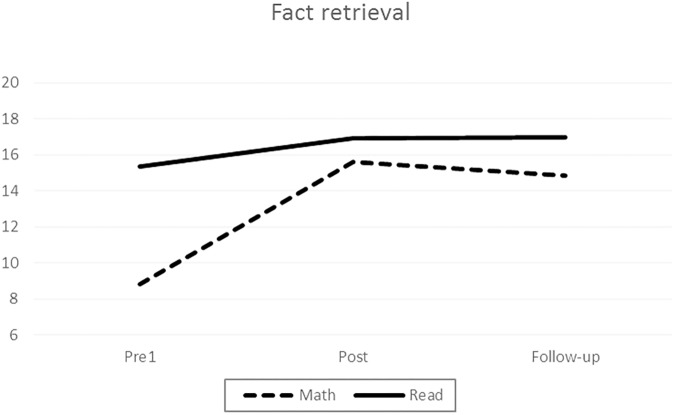
Development of fact retrieval skill.

The group–time interaction was statistically significant during the intervention (*p* < 0.001, ${\text{η}}_{\text{p}}^{2}$ = 0.31) but not during the follow-up (*p* > 0.05). As seen in **Figure 1**, the calculation fluency of the Math intervention group developed clearly during the intervention period, and their skill level remained the same during the follow-up, whereas among the Reading intervention group, small and stable improvements in calculation fluency were found throughout the entire period.

**FIGURE 2:**
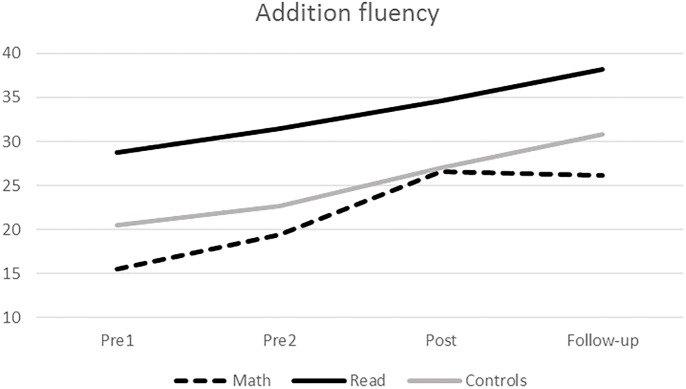
Development of addition fluency.

Second, we analyzed the *addition fluency task* using repeated-measures ANCOVA when comparing math intervention group with the reading group and ANOVA with the Business-as-usual controls. When comparing Math intervention group with Reading intervention group non-significant effect for time, gender and age were found. When comparing Math intervention group with Business-as-usual controls, there was a statistically significant main effect of time indicating that performance improved among Math intervention and Business-as-usual groups during the study period (**Tables 3**, **5**). More importantly, there was an interaction between time and group in both analyses as well, which were further explored with planned contrasts. In both analyses, the group × time interaction was statistically significant during the intervention and follow-up but not during the baseline. As seen in **Figure 2**, during the intervention period (i.e., from pre-test2 to post-test) the development of the skills of the three groups differed: although all three groups showed improvement in their skills, the Math intervention group improved faster than the other two groups and did not differ from the Business-as-usual controls at post-intervention assessment (**Table 3**). At follow-up, the fluency level remained the same in the Math intervention group, while it improved somewhat in the Reading intervention and Business-as-usual control groups. The latter groups showed a constant rate of improvement throughout the study period, while the Math intervention group showed the greatest rate of improvement during the intervention itself.

**FIGURE 3:**
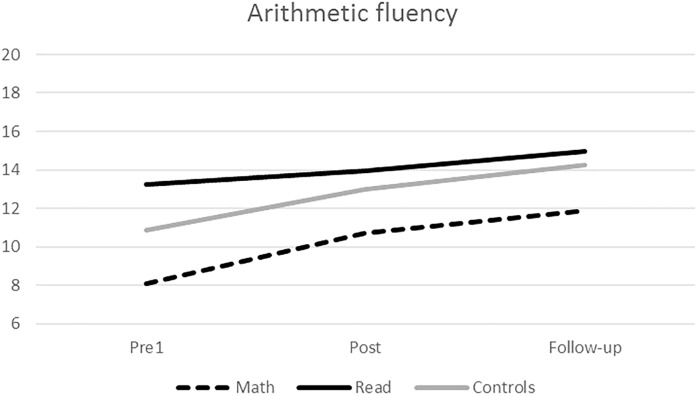
Development of arithmetic fluency.

Third, we analyzed the *arithmetic fluency task* using repeated-measures ANCOVA/ANOVA. When comparing Math intervention group with Reading intervention group non-significant effects for time, gender and age were found but significant interaction between time and group was found, suggesting that the development of arithmetic fluency differed between the Math and Reading groups. This was further explored with planned contrasts. The group × time interaction was statistically significant during the intervention but not during the follow-up. The Reading and Math intervention groups differed in their level of improvement during the intervention, but not during the follow-up. When comparing Math intervention group with Business-as-usual controls, there was a statistically significant main effect of time indicating that performance improved across Math intervention and Business-as-usual groups during the study period (**Tables 3**, **5**). In contrast, the group × time interaction was not significant compared to the Math intervention group and Business-as-usual controls. As seen in **Figure 3**, the Math intervention group and the Business-as-usual group showed similar improvement in arithmetic fluency during the intervention period, whereas the Reading intervention group showed slower improvement than the other two groups.

The Math intervention group did not show significant improvement in subtraction fluency during the intervention, and thus further analyses of progress in this subtraction fluency were not carried out.

### Changes in the Frequency of Used Strategies in the Free-Choice Condition

**FIGURE 4:**
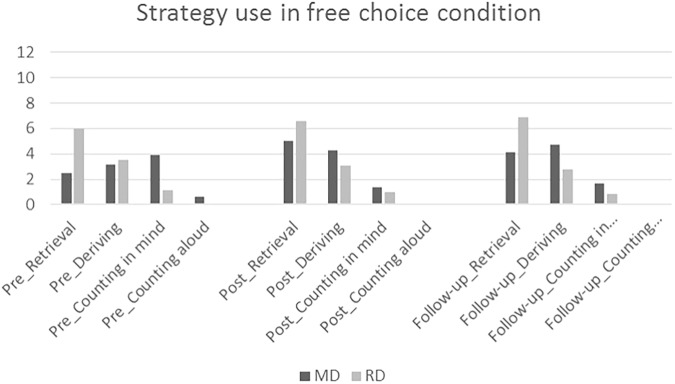
Development of strategy use in free choice condition.

Finally, the effects of the explicit strategy training on the frequency of use of different strategies were investigated. As seen in **Figure 4**, before the intervention, *counting in mind* was the most frequently used calculation strategy among the Math intervention participants, and *fact retrieval* was the most frequently used strategy among the Reading intervention group (*note*: these data were not available for the Business-as-usual controls). After the intervention, fact retrieval became the most frequently used strategy among the Math intervention participants as well; the use of *derived fact strategies* also increased in this group, while the use of *counting-based strategies* decreased among the Math intervention children. All changes during the intervention, the increasing trend in using fact retrieval and derived fact, and the decreasing trend in using counting strategies, were significant among the Math intervention children (*p* < 0.05, ${\text{η}}_{\text{p}}^{2}$ = 0.06–0.44) but not among the Reading intervention participants (*p* > 0.05) when tested using repeated measures ANCOVA. Using univariate analysis of covariance (ANCOVA) age and/or gender as the covariate it was further analyzed that the Reading intervention children used significantly more fact retrieval at each time point (*p* < 0.05), although the difference was smaller after the intervention (${\text{η}}_{\text{p}}^{2}$ = 0.03) than before the intervention (${\text{η}}_{\text{p}}^{2}$ = 0.17). There were no differences in using deriving strategies before intervention or right after (*p* < 0.05) but the Math group used more deriving strategies after 5 months follow-up (*p* < 0.05, ${\text{η}}_{\text{p}}^{2}$ = 0.04). The Math intervention group used more counting in mind strategies before the intervention (*p* < 0.05, ${\text{η}}_{\text{p}}^{2}$ = 0.10) but statistically significant differences were not found after intervention at post or follow-up assessment (*p* > 0.05). No differences (*p* > 0.05) were found in frequency of using counting aloud strategies at any assessment point, due to infrequent use of this strategy in both groups.

## Discussion

The aim of the present study was to extend the previous intervention research in math by examining whether elementary school children with poor calculation fluency benefit from strategy training focusing on derived fact strategies and following an integrative framework (i.e., integrating factual, conceptual, and procedural arithmetic knowledge). The kinds of changes in the frequency of using different strategies were also examined. The SELKIS strategy training program (Koponen et al., 2011) was implemented in small groups by trained special education teachers, highlighting the ecological validity of the present intervention study. Moreover, a 5-month follow-up was conducted to examine the long-term effects of the intervention. The results showed that children with dysfluent calculation skills participating in the Math intervention developed significantly in their addition fluency during the intervention period. They also maintained the reached fluency level during the 5-month follow-up but did not continue to further develop in addition fluency after the intensive training program ended. A similar kind of developmental slope was found both in fact retrieval as well as in addition fluency assessed in a group situation. Arithmetic fluency, covering all four arithmetical operations and both single-digit as well as multi-digit items, also improved significantly during the intervention period. In contrast, little improvement was found in subtraction fluency during the intervention, and a slightly larger but still very limited improvement was found in subtraction during the 5-month follow-up period.

Further support for a significant effect of the intervention on addition fluency comes from comparing the level of improvement in the Math intervention group with that of the two control groups. Significant group interactions were found in the forced fact retrieval task and in the addition fluency task. The Math intervention group showed more rapid improvement during the intervention than the two control groups and reached the level of Business-as-usual controls at post-intervention assessment point in addition fluency. They maintained the achieved fluency level during the 5-month follow-up but, unfortunately, did not continue to increase their calculation fluency after the intensive intervention period ended. The control groups, in contrast, showed a smooth slope of development in addition fluency throughout the period. The Math intervention group also improved significantly in arithmetic fluency during the intervention period and the interaction between time and group was significant, but their progress did not differ from that of the Business-as-usual controls. Interestingly, the Reading intervention group showed less improvement in arithmetic fluency than either of the other groups.

The maintenance of post-intervention level in addition fluency at the 5-month follow-up assessment provided support for the long-term benefits of the training. However, interesting and important question, as well, is why the Math intervention group did not continue to improve their fluency in addition after the intervention ended. There are several possible explanations. It seems that children with poor calculation fluency require explicit instruction as well as intensive training in order to extend their arithmetical knowledge and improve efficient strategy use. A summer holiday, lasting two and half months, took a place during follow-up period and naturally can be treated as a non-training period. Moreover, instruction in math typically follows periods including different mathematical contents, such as numbers and operation, geometry, and measurement etc., and thus periods focusing other contents than arithmetic, may not provide intensive training for calculation fluency. In further studies, it should be examined whether we could improve the fluency development after intensive training period by providing material and instruction how to support and strength the use of efficient calculation strategies as part of the Business-as usual instruction in a classroom.

Previous intervention research has generally assessed the efficacy of math intervention training on calculation fluency and accuracy level and not analyzed the changes in strategy use (see Fuchs et al., 2010). In the present study, in a free-choice condition math intervention children increased their use of fact retrieval and derived fact/decomposition as preferred strategies and decreased their use of counting-based strategies, which were their most common strategies before the intervention. The Math intervention group differed from the Reading group using more frequently counting-based strategies before the intervention. Differences were not significant after the intervention. Although the differences in use of retrieval strategies was significant in all assessment points favoring the Reading intervention group, the difference was clearly smaller after the intervention than before it. Moreover, the Math intervention participants used more derived fact/decomposition strategies at follow-up assessment than the Reading group. This finding suggests that despite having dysfluency in basic calculation skills after several years of training at school, explicit instruction utilizing an integrative framework in calculation strategy training can help children to use more often efficient back-up strategies and fact retrieval instead of counting.

The finding related to the missing transfer effect of addition strategy training to subtraction fluency was unfortunate. Moreover, in arithmetic fluency tasks, no developmental trend was identified among math intervention children that would have differed from their classmate control; thus, significant development in arithmetic fluency cannot be concluded to result from the intervention but could be due to schooling in general. However, this finding was not highly surprising, considering that a typical feature of children with MDs is a difficulty in spontaneously discovering efficient calculation strategies (Geary, 1993). It is likely that different arithmetical facts and arithmetic operations, e.g., addition and subtraction, are more isolated for MD children and for this reason they cannot use their knowledge of addition facts when solving subtraction problems. This could explain why spontaneous transfer did not happen across the arithmetic operations, although children started to make more frequent use of retrieval and derived fact/decomposition strategies in addition. Moreover, even typically achieving children often fail to extend their knowledge of addition principles appropriately to subtraction principles (Dowker, 1998, 2014). For example, they find the addition/subtraction inverse principle far more difficult to recognize and use than addition-specific principles, such as commutativity, and often overextend addition principles to subtraction, e.g., saying that if 14 − 5 = 9, 14 − 6 must be 10 “because 6 is one more than 5.” Thus, explicit instruction and intensive practice are likely to be required to learn to use derived fact/decomposition strategies for subtraction, rather than expecting them to spontaneously extend their strategic knowledge in addition also to subtraction.

### Limitations and Further Directions

Some limitations of the study should be considered when interpreting the current findings. The main limitations are related to the quasi-experimental nature of the design. Since the study was conducted in ecologically valid conditions as part of everyday school routines, blind and fully random matching of the participants was not achievable. However, children were carefully selected for interventions, and participants showed signs of dysfluency both in group-administered addition fluency task (where items were presented as a list) and in individually administered assessments (where items were presented one at the time). The inclusion of individual assessment is an essential strength of the participant selection, as group tests are not optimal for all children to show their abilities and could more likely lead to identifying false-positive cases. The most serious limitation may be that we did not have data from the Business-as-usual controls for all measures. However, the Reading intervention group data were available for all measures, and this is a more stringent control group.

Another limitation is that, due to the limited resources available, procedures that would allow full monitoring of the reliability and validity of the interventions (e.g., video-recordings) could not be implemented. The measures taken to guarantee the fidelity (teacher training, session-by-session manual, filling diary, meetings and phone calls during the intervention) support the understanding that the programs were implemented following the program manual and intervention design.

Finding a significant intervention effect for low-attaining children, which also remained during the follow-up period, is a positive and promising result, but at the same time only the first step. Further studies comparing this kind of integrative framework to other intervention approaches with even longer follow-up and other age groups are needed to clarify the question of what the most efficient intervention approaches for low attaining pupils are. It would also be beneficial to explore whether the intervention is equally effective in all age groups, especially given Caviola et al. (2016) findings on the differential effectiveness of derived fact training and procedural training in the third- and fifth-grade groups.

It would also be desirable to investigate the specificity of the effects, both within arithmetic and between arithmetic and other subjects. In this study, training in addition had little impact on subtraction. Further research is recommended to determine whether the same would be found regarding the effect of training in subtraction on addition.

Despite the positive findings related to the intervention effect, it should be noted that, as found in other intervention studies, there were differences in responsiveness among intervention participants. In the future, the variation in responsiveness should be studied to better understand the factors influencing the benefits of derived fact strategy training within an integrative framework, and to gain a better understanding of how to target interventions for groups of participants, and to maximize their effectiveness.

## Ethics Statement

This study was carried out in accordance with the recommendations of National Advisory Board on Research Ethics. The protocol was approved by the National Advisory Board on Research Ethics. All subjects gave written informed consent in accordance with the Declaration of Helsinki.

## Author Contributions

TK drafted a first version of the current paper. RS, AD, ER, HV, MA, and TA were responsible for draft editing.

## Conflict of Interest Statement

The authors declare that the research was conducted in the absence of any commercial or financial relationships that could be construed as a potential conflict of interest.

## References

[B1] Adetula L. O. (1996). Effects of counting and thinking strategies in teaching addition and subtraction problems. *Educ. Res.* 38 183–198. 10.1080/0013188960380206

[B2] Aro T. Viholainen H. Koponen T. Peura P. Räikkönen E. Salmi P. (in press). Can reading fluency and self-efficacy of reading fluency be enhanced with an intervention targeting the sources of self-efficacy? *Learn. Individ. Differ.*

[B3] Askew M. Bibby M. Brown M. (2001). *Raising Attainment in Primary Number Sense: From Counting to Strategy.* London: Beam Education.

[B4] Aunola K. Räsänen P. (2007). *The Basic Arithmetic Test.* Jyväskylä: Niilo Mäki Institute and University of Jyväskylä.

[B5] Bakker M. Van den Heuvel-Oppenhuizen M. Robzitsch A. (2015). Effects of mathematics computer games on special education students’ multiplicative reasoning ability. *Contemp. Educ. Psychol.* 40 55–71. 10.1016/j.cedpsych.2014.09.001

[B6] Butterworth B. Varma S. Lorillard D. (2011). Dyscalculia: from brain to education. *Science* 332 1049–1053. 10.1126/science.1201536 21617068

[B7] Bynner J. Parsons S. (1997). *Does Numeracy Matter?.* London: Basic Skills Agency.

[B8] Canobi K. H. (2005). Children’s profiles of addition and subtraction understanding. *J. Exp. Child Psychol.* 92 220–246. 10.1016/j.jecp.2005.06.001 16024038

[B9] Caviola S. Gerotto G. Mammarella I. (2016). Computer-based training for improving mental calculation in third- and fifth-graders. *Acta Psychol.* 171 118–127. 10.1016/j.actpsy.2016.10.005 27794217

[B10] Chodura S. Kuhn J.-T. Holling H. (2015). Interventions for children with mathematical difficulties: a meta-analysis. *Z. Psychol.* 223 129–144. 10.1027/2151-2604/a000211

[B11] Christensen C. A. Gerber M. M. (1990). Effectiveness of computerized drill and practice games in teaching basic math facts. *Exceptionality* 1 149–165. 10.1080/09362839009524751

[B12] Cirino P. T. Ewing-Cobbs L. Barnes M. Fuchs L. S. Fletcher J. M. (2007). Cognitive arithmetic differences in learning disability groups and the role of behavioral inattention. *Learn. Disabil. Res. Pract.* 22 25–35. 10.1111/j.1540-5826.2007.00228.x

[B13] Dowker A. (1998). “Individual differences in arithmetical development,” in *The Development of Mathematical Skills*, ed. Donlan C. (London: Psychology Press), 275–302.

[B14] Dowker A. (2009). Derived fact strategies in children with and without mathematical difficulties. *Cogn. Dev.* 24 401–410. 10.1016/j.cogdev.2009.09.005 22717148

[B15] Dowker A. (2014). Young children’s use of derived fact strategies for addition and subtraction. *Front. Hum. Neurosci.* 7:924. 10.3389/fnhum.2013.00924 24431996 PMC3880841

[B16] Dowker A. (2017). Interventions for primary school children with difficulties in mathematics. *Adv. Child Dev. Behav.* 53 255–287. 10.1016/bs.acdb.2017.04.004 28844246

[B17] Dowker A. Sigley G. (2010). Targeted interventions for children with arithmetical difficulties. *Br. J. Educ. Psychol.* 7 65–81. 10.1348/97818543370009X12583699332492

[B18] Fuchs L. Fuchs D. Hamlet C. L. Powell S. R. Capizzi A. M. Seethaler P. M. (2006). The effects of computer-assisted instruction on number combination skill in at-risk first graders. *J. Learn. Disabil.* 39 467–475. 10.1177/00222194060390050701 17004677

[B19] Fuchs L. S. Powell S. R. Hamlett C. L. Fuchs D. Cirino P. T. Fletcher J. M. (2008). Remediating computational deficits at third grade: a randomized field trial. *J. Res. Educ. Effect.* 1 2–32. 10.1080/19345740701692449 21709759 PMC3121170

[B20] Fuchs L. S. Powell S. R. Seethaler P. M. Cirino P. T. Fletcher J. M. Fuchs D. (2009). Remediating number combination and word problem deficits among students with mathematics difficulties: a randomized control trial. *J. Educ. Psychol.* 101 561–576. 10.1037/a0014701 19865600 PMC2768320

[B21] Fuchs L. S. Powell S. R. Seethaler P. M. Cirino P. T. Fletcher J. M. Fuchs D. (2010). The effects of strategic counting instruction, with and without deliberate practice, on number combination skill among students with mathematics difficulties. *Learn. Individ. Diff.* 20 89–100. 10.1016/j.lindif.2009.09.003 20383313 PMC2850218

[B22] Geary D. C. (1993). Mathematical disabilities: cognitive, neuropsychological, and genetic components. *Psychol. Bull.* 114 345–362. 10.1037/0033-2909.114.2.345 8416036

[B23] Geary D. C. (2004). Mathematics and learning disabilities. *J. Learn. Disabil.* 37 4–15. 10.1177/00222194040370010201 15493463

[B24] Geary D. C. Hamson C. O. Hoard M. K. (2000). Numerical and arithmetical cognition: a longitudinal study of process and concept deficits in children with learning disability. *J. Exp. Child Psychol.* 77 236–263. 10.1006/jecp.2000.2561 11023658

[B25] Geary D. C. Hoard M. K. Bailey D. H. (2012). Fact retrieval deficits in low achieving children and children with mathematical learning disability. *J. Learn. Disabil.* 37 291–307. 10.1177/0022219410392046 21252374 PMC3163113

[B26] Geary D. C. Hoard M. K. Byrd-Craven J. Nugent L. Numtee C. (2007). Cognitive mechanisms underlying achievement deficits in children with mathematical learning disability. *Child Dev.* 78 1343–1359. 10.1111/j.1467-8624.2007.01069.x 17650142 PMC4439199

[B27] Gersten R. Jordan N. C. Flojo J. R. (2005). Early identification and interventions for children with mathematics difficulties. *J. Learn. Disabil.* 28 293–304. 10.1177/00222194050380040301 16122059

[B28] Girelli L. Bartha L. Delazer M. (2002). Strategic learning in the rehabilitation of semantic knowledge. *Neuropsychol. Rehabil.* 12 41–61. 10.1080/09602010143000149

[B29] Gross J. Hudson C. Price D. (2009). *The Long- Term Costs of Numeracy Difficulties.* London: Every Child a Chance Trust (KPMG).

[B30] Hasselbring T. Goin L. Bransford J. (1988). Developing math automaticity in learning handicapped children: the role of computerized drill and practice. *Focus Except. Children* 20 1–7. 10.17161/fec.v20i6.7504

[B31] Holmes W. Dowker A. D. (2013). Catch Up Numeracy: a targeted intervention for children who are low attaining in mathematics. *Res. Math. Educ.* 15 249–265. 10.1080/14794802.2013.803779

[B32] Jordan N. C. Hanich L. B. Kaplan D. (2003). A longitudinal study of mathematical competencies in children with specific mathematics difficulties versus children with comorbid mathematics and reading difficulties. *Child Dev.* 74 834–850. 10.1111/1467-8624.00571 12795393 PMC2791887

[B33] Jordan N. C. Montani T. O. (1997). Cognitive arithmetic and problem solving: a comparison of children with specific and general mathematics difficulties. *J. Learn. Disabil.* 30 624–634. 10.1177/002221949703000606 9364900

[B34] Kaufmann L. Mazzocco M. M. Dowker A. Von Aster M. Göbel S. M. Grabner R. H. (2013). Dyscalculia from a developmental and differential perspective. *Front. Psychol.* 4:516. 10.3389/fpsyg.2013.00516 23970870 PMC3748433

[B35] Koponen T. Aro T. Ahonen T. (2009). Conceptual knowledge-based strategy training in single-digit calculation: a single case intervention study. *Eur. J. Special Needs Educ.* 24 259–276. 10.1080/08856250903016813

[B36] Koponen T. Mononen R. Kumpulainen T. Puura P. (2011). *SELKIS-Yhteenlaskua Ymmärtämään. Yhteenlaskutaidon Harjoitusohjelma. [Improving Addition Skills: SELKIS Intervention Program].* Jyväskylä: Niilo Mäki Institute and Haukkarannan koulu.

[B37] Parsons S. Bynner J. (2005). *Does Numeracy Matter More?.* London: NRDC.

[B38] Powell S. R. Fuchs L. S. Fuchs D. Cirino P. Fletcher J. M. (2009). Effects of fact retrieval tutoring on third-grade students with math difficulties with and without reading difficulties. *Learn. Disabil. Res. Pract.* 24 1–11. 10.1111/j.1540-5826.2008.01272.x 19448840 PMC2682421

[B39] Raven J. Court J. H. Raven J. C. (1999). Manual for Raven’s Progressive Matrices and Vocabulary Scales - Section 2: Coloured Progressive Matrices. Oxford: Oxford Psychologists Press.

[B40] Russell R. Ginsburg H. P. (1984). Cognitive analysis of children’s mathematical difficulties. *Cogn. Instr.* 1 217–244. 10.1207/s1532690xci0102_3

[B41] Shalev R. S. Manor O. Gross-Tsur V. (2005). Developmental dyscalculia: a prospective six-year follow-up. *Dev. Med. Child Neurol.* 47 121–125. 10.1111/j.1469-8749.2005.tb01100.x 15707235

[B42] Siegler R. S. (1996). *Emerging Minds: The Process of Change in Children’s Thinking.* New York, NY: Oxford University Press.

[B43] Siegler R. S. Shrager J. (1984). “Strategy choice in addition and subtraction: How do children know what to do?,” in *Origins of Cognitive Skills*, ed. Sophian C. (Hillsdale, NJ: Erlbaum), 229–293.

[B44] Star J. R. Rittle-Johnson B. (2008). Flexibility in problem solving: the case of equation solving. *Learn. Instr.* 18 565–579. 10.1063/1.4834638 24320369

[B45] Steinberg R. (1985). Instruction in derived facts strategies in addition and subtraction. *J. Res. Math. Educ.* 16 337–355. 10.2307/749356

[B46] Thornton C. A. (1978). Emphasizing thinking strategies in basic fact instruction. *J. Res. Math. Educ.* 9 214–227. 10.2307/748999

[B47] Torgerson C. J. Wiggins A. Torgerson D. J. Ainsworth H. Barmby P. Hewitt C. (2011). *Every Child Counts: The Independent Evaluation Executive Summary.* London: Department for Education (DfE).

[B48] Tournaki N. (2003). The differential effects of teaching addition through strategy instruction versus drill and practice to students with and without learning disabilities. *J. Learn. Disabil.* 36 449–458. 10.1177/00222194030360050601 15497488

[B49] Wechsler D. (2010). *WISC-IV – Wechsler Intelligence Scale for Children—IV.* Helsinki: Psykologien Kustannus.

[B50] Woodward J. (2006). Developing Automaticity in Multiplication Facts: integrating strategy instruction with timed practice drills. *J. Disabil. Q.* 40 270–281. 10.2307/30035554

